# Biperiden Dependence: Case Report and Literature Review

**DOI:** 10.1155/2012/949256

**Published:** 2012-05-30

**Authors:** Fernando Espi Martinez, Fernando Espi Forcen, Arlenne Shapov, Amparo Martinez Moya

**Affiliations:** ^1^Department of Internal Medicine, University of Murcia, 30120 Murcia, Spain; ^2^Department of Psychiatry, MetroHealth Medical Center, Case Western Reserve University, Cleveland, OH 44109, USA; ^3^Department of Psychiatry, Hospital Virgen de La Arrixaca, University of Murcia, 30120 Murcia, Spain

## Abstract

Anticholinergic drugs are frequently used in psychiatry for the prophylaxis and treatment of extrapiramidal symptoms caused by neuroleptics. Abuse of anticholinergic agents has been reported in patients with psychotic disorders, on treatment with neuroleptics, and polysubstance use disorders. We are reporting the case of a patient who presented with hypoactive delirium as a consequence of biperiden dependence. The clinician must pay special attention to detect anticholinergic misuse in patients presenting with delirium of unknown cause.

## 1. Introduction

Anticholinergic drugs such as biperiden, benztropine, procyclidine, and trihexyphenidyl are regularly used in clinical practice for the prophylaxis and treatment of extrapiramidal side effects associated with neuroleptics as well as for tremors in Parkinson's disease [1].

Although thought to be rare, anticholinergic misuse has been reported in several clinical settings. Its abuse potential could be related to its inhibiting action on neuroleptic-induced anhedonia [2–4]. Also, several cases of polysubstance abuse including biperiden have been reported [5, 6].

The neurotransmitter acetylcholine is thought to play a major role in the pathogenesis of delirium. This was first proposed when electroencephalographic changes compatible with delirium were found after the administration of anticholinergic substances and reversal with cholinergic agents. Anticholinergic delirium may present with altered mental status, waxing and waning level of consciousness, urinary retention, attention and concentration deficits, dry mouth, cardiac arrhythmia, blurred vision, hypothermia, fever, hot skin, and decreased convulsive threshold [1, 7, 8].

We are reporting a case of a patient who came to the addiction unit for treatment of biperiden dependence. He initially presented hypoactive delirium associated to his anticholinergic use.

## 2. Case Report

This is a 47-year-old-man that was referred to the addiction unit of Hospital de Virgen de La Arrixaca in Murcia, Spain for the treatment of anticholinergic drug abuse. Patient reported self-medicating with biperiden and developing tolerance to this drug. He described this effect as feeling relaxed and sociable. Given his multiple unsuccessful attempts to quit biperiden, the patient thought that he was addicted to this drug and wanted to be treated for it.

Past psychiatric history was significant for a 31-year history of alcohol and marijuana abuse and a 22-year history of cocaine abuse. At the time of presentation, he had been abstinent from alcohol or other drugs for two years. Family history was significant for a brother with alcoholism and an uncle who committed suicide. The patient had been hospitalized 18 times for behavioral problems related to his substance abuse. He was taking pregabalin 75 mg twice daily and 5 mg of diazepam every 8 hours as needed for anxiety as prescribed by his family physician.

On his first visit to our unit, the patient had already been using biperiden for three months. He had progressively increased the amount and was taking up to 50 mg daily. He presented with an anticholinergic intoxication. He was somnolent and disoriented to time and place. He had poor short-term memory and showed generalized weakness. He had poor and incoherent speech and was not able to carry a normal conversation. He complained of dry mouth, blurry vision, resting tremor, urinary incontinence, constipation, and poor appetite. Family reported that the patient had seemed very confused and was not able to do simple tasks such as eating at the table. On further questioning, the patient admitted to endorsing illusions, as he felt that objects seemed to change shape when looking at them. At times he would also become psychotic, as he described feeling that someone was mumbling things at him.

Physical exam was significant for generalized weakness, hypotonia, and hyporreflexia without focal neurological signs, flaccidity in the facial muscles, and slow gait. Orthostatic vital signs were taken: heart rate was 90 and 95 b.p.m. Blood pressure was 100/75 and 85/70. Complete blood count, basic metabolic panel, thyroid hormones, and liver function tests were within normal limits. Urine toxicology was positive for benzodiazepines only. CT scan of the head, abdominal ultrasound, EKG and chest X-ray did not show abnormalities.

A Mini-Mental Status Exam (MMSE) could not be performed due to the severity of his confusion. Our patient was treated in an outpatient setting for acute anticholinergic delirium. Biperiden was discontinued and diazepam increased to 10 mg every 8 hours as needed for three weeks. Pregabalin was continued. Family monitored his behavior at home to avoid falls. Eight days after the first visit the patient came back to the unit and admitted to continue using 6–8 mg of biperiden daily. He reported withdrawal symptoms such as anxiety, restlessness, and insomnia. A cognitive evaluation was still not possible because he remained very confused. Patient was reevaluated 10 days later. At that time MMSE score was 20/30. Two weeks later he was seen again and his MMSE was 23/30. In both cognitive evaluations his short-term memory was significantly impaired. These results are consistent with mild cognitive deterioration most likely associated with biperiden overuse as well as a previous history of chronic alcohol use.

Five months after the first visit the patient was seen again in the office. Patient had stopped using after the previous visit but had relapsed three weeks prior. He was taking 30 mg daily. He presented with similar but milder symptoms than the first visit. He was again treated with diazepam 10 mg every 8 hours as needed and Motivational Interviewing Therapy.

Seven months after the first visit, Millon Multiaxial Clinical Inventory (MMCI-III) was applied and results were not consistent with a baseline personality disorder.

Twelve months after the first visit patient was seen again. He denied cravings and had been abstinent from biperiden and any other drug for seven months.

## 3. Discussion

To our knowledge this is the first case of hypoactive delirium in a patient with isolated biperiden dependence. The presence of attention deficits and memory disturbances supports a diagnosis of delirium rather than a substance-induced psychotic disorder [9]. The patient suffered from a baseline mild cognitive deterioration that was probably related to his previous alcohol and drug use. His urinary incontinence was probably related to an excessive urinary retention caused by biperiden.

Since symptoms remitted after removing the offending agent, and he experienced similar effects after relapsing on biperiden, the possibility that our patient suffered a primary episode of delirium is low. The patient developed tolerance, withdrawal, and relapse, which is consistent with biperiden dependence. It is difficult to determine the exact moment that biperiden dependence started. However, the day of his first visit he already met the following four out of the seven diagnostic DSM-IV-TR criteria for substance dependence: he showed tolerance since he had been increasing the dose up to 50 mg daily to achieve the desired effects (criteria 1). He was taking biperiden in such an amount that he developed anticholinergic delirium (criteria 3). He was reporting unsuccessful efforts to cut down (criteria 4). Due to his substance use he was failing to complete daily chores (criteria 6). At the time of the first visit to our clinic the patient already met DSM-IV-TR criteria for substance dependence. When biperiden was discontinued he experienced withdrawal symptoms (criteria 2) and continued to use it until the second visit. Despite having suffered an anticholinergic delirium due to biperiden misuse he relapsed four months later (criteria 7).

 Physostigmine was not used for the treatment of the patient's anticholinergic intoxication to avoid dangerous side effects such as cholinergic crisis, seizures, and asystolia. Benzodiazepines and behavior monitoring are considered the best alternative to physostigmine [10], and therefore were used in this case.

The prevalence of anticholinergic misuse remains unclear. Burich reported a 34% prevalence of anticholinergic misuse in a sample of 50 patients suffering from chronic psychotic disorders [5]. A study done in Jordan stated that anticholinergic substances were the most frequently abused after opiates, cocaine, marijuana, and amphetamines [11]. Gjerden et al. found that among patients that were not on neuroleptics nor had Parkinson's disease, biperiden was the anticholinergic most frequently used concomitant with benzodiazepines. This could explain that our patient, who while taking diazepam, used biperiden over other anticholinergic substances [12].

Biperiden and other anticholinergic agents are potential drugs of abuse. This can be explained by their ability to ameliorate negative psychotic symptoms as well as their inhibition of neuroleptic induced anhedonia [1]. As a result patients on neuroleptics are at risk of abusing anticholinergic agents such as biperiden. The second population at risk involves those with significant history of polysubstance use just like our patient [7]. In a double-blind control placebo study biperiden was found to elevate mood in healthy subjects [13]. This could explain biperiden's potential for abuse.

There is still not a good biochemical theory that explains anticholinergic dependence. It is well known that the mesolimbic dopaminergic system formed by the ventral tegmental area, the nucleus accumbens, and the prefrontal cortex is the common final pathway for the reinforcing effect of drug abuse [14]. The cholinergic system may play a role in drug addiction. Activation of muscarinic receptors can facilitate dopaminergic transmission and release in the nucleus accumbens. By blocking muscarinic receptors, biperiden may inhibit dopamine reuptake and storage. This could explain its euphoric action as well as its deliriogenic effects [15].

In patients presenting with delirium of unknown cause, the clinician must pay special attention to detect anticholinergic drug misuse. Patients with a history of chemical dependence or treatment with neuroleptics might be at special risk.
